# Are patients with Parkinson's disease impaired in the recognition of emotion's authenticity?

**DOI:** 10.1111/jnp.12410

**Published:** 2024-12-26

**Authors:** Agnese Anzani, Stefano Zago, Teresa Difonzo, Cristina Scarpazza, Nadia Bolognini, Giulia Franco, Alessio Difonzo, Maria Cristina Saetti

**Affiliations:** ^1^ Department of Pathophysiology and Transplantation University of Milan Milan Italy; ^2^ Foundation IRCCS Ca' Granda Ospedale Maggiore Policlinico Neurology Unit Milan Italy; ^3^ Department of General Psychology University of Padova Padova Italy; ^4^ IRCCS S Camillo Hospital Venezia Italy; ^5^ Department of Psychology and NeuroMI‐Milan Centre for Neuroscience University of Milano‐Bicocca Milan Italy; ^6^ Laboratory of Neuropsychology, Department of Neurorehabilitation Sciences IRCCS Istituto Auxologico Italiano Milan Italy

**Keywords:** embodied cognition, emotion authenticity, facial emotion recognition, human mirror system, Parkinson's disease

## Abstract

In recognising emotions expressed by others, one can make use of both embodied cognition and mechanisms that do not necessarily require activation of the limbic system, such as evoking from memory the meaning of morphological features of the observed face. Instead, we believe that the recognition of the authenticity of an emotional expression is primarily based on embodied cognition, for which the mirror system would play a significant role. To verify this hypothesis, we submitted 20 parkinsonian patients and 20 healthy control subjects to the Emotional Authenticity Recognition test, a novel test using dynamic stimuli to evaluate the ability to recognise emotions and their authenticity. Analysis of variance of the test scores shows that Parkinsonian patients perform worse than controls when they had to recognise the authenticity of emotions, although they are able to identify them. Our results confirm a deficit in the recognition of the authenticity of emotions in patients with Parkinson's disease attributable to the disruption of extrapiramidal limbic circuit between ventral striatum and orbitomesial‐prefrontal cortex.

## INTRODUCTION

Recognising emotions expressed by others is one of the crucial abilities involved in the process of social perception (Elfenbein et al., 2007). It belongs to the broader spectrum of cognitive abilities referred to as social cognition (Frith, 2008), together with the theory of mind, empathy, and social behaviour. Through these cognitive processes, it is possible to recognise the emotional state of others, to understand what they may be feeling in order to predict their behaviour and guide one's responses accordingly (Beaudoin & Beauchamp, 2020).

The mechanism of embodied cognition is believed to be the process at the core of recognising emotions. That mechanism refers to the activity of re‐experiencing perceptual, somatovisceral and motoric stimuli related to an emotion by past events, while perceiving or thinking about it (Goldman & Sripada, 2005; Niedenthal, 2007; Pitcher et al., 2008; Wood et al., 2015). A central role in this process could be played by the mirror neurons system, which does activate both when a specific action is performed and when the same action is seen in others, as well as when a specific stimulus is perceived and when its perception is observed in others (Bolognini et al., 2012; Bolognini et al., 2014; Keysers et al., 2010; Rizzolatti & Sinigaglia, 2016). It allows an implicit understanding of the behaviour, intention and feelings of others, and it seems to be involved in most of the functions of social cognition (Ferrari & Coudé, 2018).

Just as the mirror system allows us to understand the intention of other individuals by observing their goal‐directed actions, it could also allow us to identify the emotions of others by observing their facial expression. The hypothesis is supported by neuropsychological studies showing a correlation between emotion expression and recognition abilities both in normal subjects (Ricciardi et al., 2017) and in patients with extrapyramidal diseases (Ricciardi et al., 2017; Trinkler et al., 2013), and by functional magnetic resonance imaging (fMRI) studies (Carr et al., 2003) showing a marked overlap between the regions activated by observation and imitation of emotional facial expressions. However, the hypothesis is contradicted by the demonstration that patients with Moebius syndrome do not have facial expression recognition disorders (Rives Bogart & Matsumoto, 2010), suggesting that the recognition of emotion can also rely on semantic knowledge (Folstein et al., 2013; Smith et al., 2005; Vannuscorps et al., 2020).

Facial emotion recognition has been studied among several groups of neurological patients, particularly those with neurodegenerative conditions (Stam et al., 2023; Yitzhak et al., 2020). We will focus on patients affected by Parkinson's Disease (PD), in which facial emotion recognition has been widely analysed, as reviewed by Gray and Tickle‐Degnen (2010), Péron et al. (2012), Argaud et al. (2018), and, more recently, by Coundouris and colleagues (2020).

Most of the studies indicate an impairment of emotion recognition in PD patients. This would be due to dopamine depletion in the mesolimbic pathway, which is responsible for a disruption of the connection between the basal ganglia and the orbitofrontal and cingulate cortex (Lin et al., 2016; Péron et al., 2012).

The facial emotion recognition impairment of PD would support the theory of embodied simulation, according to which recognition of an expression is facilitated by its simulation, which activates internal somatosensory representations and corresponding emotional states in the observer. Indeed, the simulation of the observed expression is impaired in the case of hypomimia. The role of the mirror system in this process is supported by fMRI studies, which point out that the deactivation of frontal and parietal human homologues of mirror neuron areas in PD can lead to a disruption of neural resonance and thus underlie impaired emotion recognition (Pohl et al., 2017).

Nevertheless, it should be noted that only 64% (Argaud et al., 2018) and 75% (Gray & Tickle‐Degnen, 2010) of the studies have reported an actual impairment in facial emotion recognition compared to healthy controls. In order to explain the discrepancies in the results, several confounding factors, both clinical (patient characteristics such as duration and severity of the disease, medication, cognitive deficits, mood disorders) and methodological (dynamism and emotional intensity of the facial expression stimuli, difficulty and sensitivity of the task) have been highlighted (Argaud et al., 2018). Of relevance is the possible presence of concomitant cognitive deficits, such as reduced ability to perceive visual forms (Marneweck et al., 2014; Narme et al., 2011) and to consciously process visual stimuli (Alonso‐Recio et al., 2014; Dujardin et al., 2013), which are quite common in PD, due to neuronal loss in certain striatal and cortical regions. Therefore, it is currently unclear whether the disturbance of facial emotion recognition in PD means loss of affective sharing and empathy, or whether it is collateral to a disturbance of perceptual processing of visual stimuli leading to their recognition.

It has been hypothesised that the rational recognition of emotions may also be based on semantic knowledge (Bertoux et al., 2020). For example, one recognises the expression of happiness because it is known that the upward angles of the lips and the raised cheeks represent a smile. This idea is supported by data on patients with semantic variant of frontotemporal dementia (Bertoux et al., 2020). The semantic strategy can be used when the relevant facial features are easily perceived. Indeed, the identification of emotions can be considered an easy task when their expressions are sufficiently intense and not confused (Straulino et al., 2023).

We assume that facial emotion recognition occurs simultaneously through two mechanisms, embodied cognition and visual‐perceptive/semantic processing, and that under pathological conditions one can supplant the other either more or less efficiently.

One of the most important aspects of communication and social interaction lies in the perceived authenticity of the expressed emotion (Lu et al., 2020; Rooney et al., 2012). Emotions can be ‘authentic’, that is, generated by genuine experiences (Deckert et al., 2020; Lu et al., 2020; Rooney et al., 2012; Zloteanu & Krumhuber, 2021), or ‘simulated’, if they do not reflect true feeling, but they allow for strategic advantages (Ekman & Rosenberg, 2005; Monaro et al., 2022). Posed emotion are often prototypical and very intense (Barrett et al., 2019), while authentic emotions can occur in millisecond (e.g., micro‐expressions) and are often less intense and more subtle (Tcherkassof et al., 2013). When individuals are asked to assess the authenticity of emotion on the basis of subtle facial features, the semantic strategy can be extremely difficult, or even impossible, to apply (Niedenthal et al., 2010).

We assume that the ability to distinguish the authenticity of emotional expressions would almost entirely require embodied simulation and mirror system activation.

The emotional stimuli classically used in research, as well as implemented in clinical tests, depict faces where emotions are posed and statically depicted. Examples of these tests include the Ekman 60 faces test emotion labelling and emotion discrimination (Ekman & Friesen, 1976; Young et al., 2002), the Reading the Mind in the Eye (Baron‐Cohen et al., 2001) the FACE test (Terruzzi et al., 2023), the Geneva Emotion Recognition test (Schlegel et al., 2014). However, posed expressions do not allow an assessment of the ability to recognise the authenticity of the emotion. Moreover, if the static nature of stimuli interferes with the accuracy of emotion recognition in real life situation (Argaud et al., 2018), it interferes even more with the recognition of emotion authenticity, based on very rapid and nuanced micro‐expressions (Straulino et al., 2023).

Our work aims to study facial emotion recognition among PD, using the Emotional Authenticity Recognition (EAR) test (Gramegna et al., 2023; Scarpazza et al., 2024), an innovative test using dynamic stimuli to evaluate the ability to recognise emotions and emotions' authenticity selected from the Padova Emotional Dataset of Facial Expressions (PEDFE) (Miolla et al., 2023). The EAR test allows us to make new assumptions regarding cognitive processing and neural pathways involved in this ability. We expect that cognitive uncompromised PD patients will be mainly impaired in recognising emotion's authenticity rather than in recognising emotions themselves. This could be explained by the dysfunction of the limbic orbito‐mesial extrapyramidal circuit, which may precede dorsolateral cognitive circuit involvement in disease progression.

## METHODS

### Participant sample

Twenty PD patients and 20 healthy control subjects (HC) were included to the study. All participants were Caucasian, Italian native‐speakers and right‐handed according to the Edinburgh Handedness Inventory (Oldfield, 1971). Additional demographic characteristics of the participants are shown in Table 1.

**TABLE 1:** Participants' demographic features and between groups comparison. Standard deviations are reported in parenthesis.

	HC	PD	*χ* ^2^ or F	Df	*p*
Sex at birth (M/F)	9/11	12/8	.714	1	ns
Mean age (years)	65.7 (12.3)	65.8 (12.5)	.038	38	ns
Mean education (years)	12.4 (3.5)	12.6 (3.3)	.184	38	ns

Abbreviations: F, female; HC, healthy control subjects; M, male; PD, individuals with Parkinson's disease.

The total number of recruited PD patients was 20:12 males and eight females (mean age = 65.7 ± 12.5; mean years of schooling 12.4 ± 3.3).

Patients who met the classification criteria of PD according to UK Parkinson's Disease Brain Bank (Cartabellotta et al., 2018) were recruited over a six‐month period at the Movement Disorders Center, Neurology U.O.C. of the ‘Policlinico Maggiore’ Hospital, in Milan. Subjects affected by atypical parkinsonism (multiple system atrophy, progressive supranuclear palsy, etc.) and secondary parkinsonism were not included. Motor disability was assessed via the third section of the Unified Parkinson's Disease Rating Scale (UPDRS‐III). Only patients in the mild and moderate stages of the disease (Martínez‐Martín et al., 2015) were selected—based on the assumption that the degree of cognitive impairment tends to parallel the degree of the motor involvement (Dubois et al., 1992). All PD patients were treated with standard medications and were tested in ON phase. The mean values (standard deviations) of UPDRS‐III and Levodopa equivalent daily dose (LEDD) are shown in Table 2.

**TABLE 2:** Patients' clinical and cognitive features. Standard deviations are reported in parenthesis.

PD (*n* = 20)	Mean (SD)	Range
Disease duration (years)	6.05 (5.91)	1–28
UPDRS‐III	15.35 (8.09)	4–31
LEDD (mg)	581.85 (346.61)	126–1295
MoCA (correct score)	24.7815 (2.89)	17.52–28.65
BFRT (correct score)	43.75 (5.17)	37–53

Abbreviations: BFRT, Benton Facial Recognition Test; LEDD, Levodopa Equivalent Daily Dose; MoCA, Montreal Cognitive Assessment; SD, Standard Deviation; UPDRS‐III, Unified Parkinson's Disease Rating‐Scale‐third section.

The exclusion criteria for the study were: suspected clinical cognitive decline or depression; history of any other significant neurological or psychiatric disorders; non‐neurological comorbidities that could potentially compromise neuropsychological testing results; use of psychotropic drugs. Therefore, patients underwent a thorough cognitive‐behavioural history and a psychiatric interview. Only patients without any clinical suspicion of dementia, without symptoms of depression and who did not require psychotropic pharmacological therapy were selected. Furthermore, to rule out cognitive decline, patients had to score at least 15/30 on the Montreal Cognitive Assessment (MoCA) (Conti et al., 2015). We preferred the use MoCA over MMSE, because several studies have underlined a better sensibility of this tool in the evaluation of cognitive decline in PD (D'Iorio et al., 2023; Hoops et al., 2009; Zadikoff et al., 2008). Beyond the exclusion of cognitive decline, to rule out visuo‐perceptive disorders interfering with face processing, patients had to score at least 37/45 on the Benton Facial Recognition Test (BFRT) (Albonico et al., 2017). The mean scores for MoCA and BFRT, their standard deviations and the range of scores obtained by the patients are reported in Table 2. Finally, available imaging data (CT or MRI) had to result negative for brain alterations.

Informed written consent was acquired from all patients before the evaluation and the study protocol was reviewed and approved by the Milan Area 2 Ethics Committee (ID 80962), in compliance with the World Medical Association Declaration of Helsinki.

Twenty HC, matched to PD patients for age, gender and years of education, took part in the experiment.

The control subjects were chosen from a larger group tested at Università degli Studi di Padova. Subjects had to have no neurological or psychiatric disorders and, if they were over 50 years old, had to score higher than 15/30 on the MoCA (Conti et al., 2015). They took part in this study on a voluntary basis, after having provided their written informed consent. The study was approved by the Ethics Committee of the University of Padova (protocol number: 3954), in compliance with the guidelines of the Helsinki Declaration (1975).

### Emotion authenticity recognition test

**FIGURE 1:**
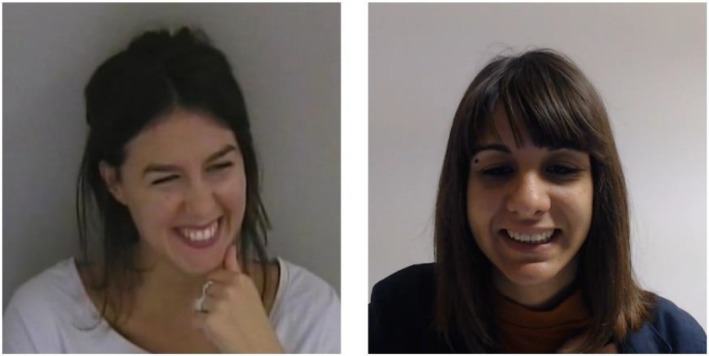
An example of emotion recognition test stimuli: Happiness expression, authentic (left) and posed (right).

**FIGURE 2:**
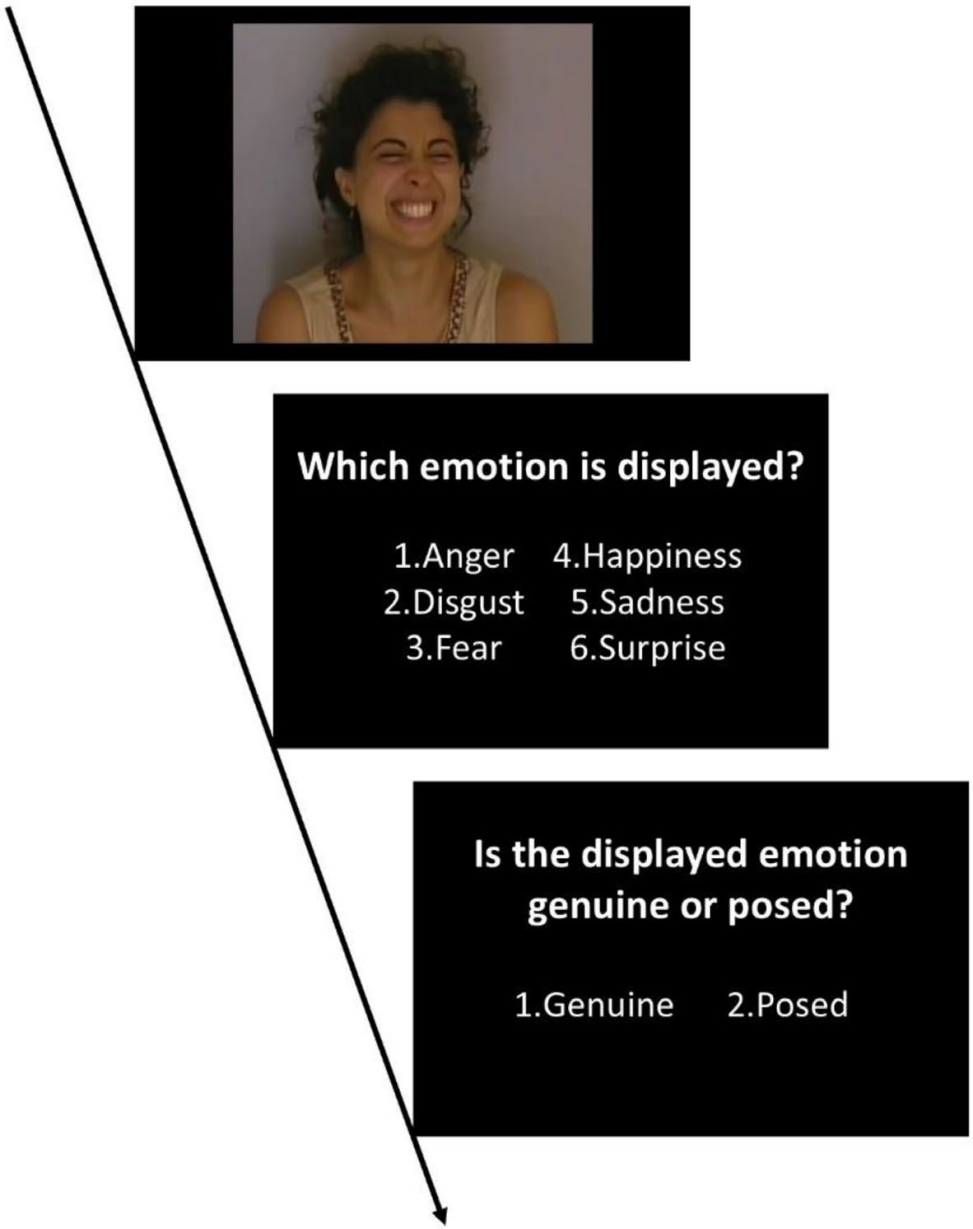
Experimental procedure of the emotion recognition test: Emotion presentation (e.g., posed happiness) and experimental questions.

The facial emotion recognition process was studied using the Emotional Authenticity Recognition (EAR) test (Gramegna et al., 2023). Developed in 2023 to expand the tools available to investigate emotion‐related processes, it consists of short videos of faces expressing the six basic emotions (happiness, sadness, fear, surprise, anger and disgust). Facial expressions belong to subjects filmed both when conveying a genuine emotional reaction induced by visual or non‐visual input and when acting it out (see Figure 1). The validated (Scarpazza et al., 2024) neuropsychological test consists of 60 clips of 30 authentic emotions and 30 acted emotions, contained in a Power Point file. The participants of our study were asked to recognise the portrayed emotion of each clip and to specify if it was real or acted (see Figure 2). It was possible for them to watch the videos twice.

Two main parameters were used to evaluate subjects' performance: the Emotion Recognition Index (ERI), that is, the number of correct answers identifying the type of emotion, and the Emotion Authenticity Index (EAI), the number of correct answers identifying the spontaneity of the emotions. The maximum score for each of the two index is 60. The EAR test does not provide individual scores for the different emotions.

### Statistical analyses

Analyses were run via jamovi 2.3 (https://www.jamovi.org).

A bivariate analysis of covariance was performed to study the group effect on ERI and EAI, taking age, education and sex at birth as covariates. Normality assumptions, as tested on residuals, were satisfied by the model, graphical (i.e., no outliers in the Q–Q graph) and inferential levels (i.e., the Shapiro–Wilk's statistic is not significant if the significance level was set at *α* = .01).

Within PD patients, the relationship between each of the two variables expressing emotion recognition (ERI, EAI) and the set of variables expressing clinical or cognitive features of the disease (UPDRS‐III, disease duration, LEDD, MoCA score and BFRT score) was studied with two separate multiple linear regression analyses. For the regression analyses, both ERI and EAI were adjusted for age and sex (Scarpazza et al., 2024).

## RESULTS

The mean and standard deviation of ERI and EAI obtained from PD and HC are shown in Table 3, the results of the bivariate analysis of covariance are shown in Table 4. A significant main effect of Group (F = 3.40, df = 2, 34 *p* = .045) was detected. Compared to HC, PD patients scored lower EAI (F = 6.320, df = 1, *p* = .0017). However, ERI did not differ significantly between the groups (F = 3.982, df = 1, *p* = .054), although PD showed a tendency to perform worse.

**TABLE 3:** Mean values (standard deviations) of emotion and authenticity recognition scores achieved by individuals with Parkinson's disease and healthy control subjects.

	HC	PD
ERI	47.6 (5.99)	44.5 (7.46)
EAI	47.3 (5.52)	43.5 (6.58)

Abbreviations: EAI, emotion authenticity index; ERI, emotion recognition index; HC, healthy control subjects; PD, individuals with Parkinson's disease.

**TABLE 4:** Bivariate analysis of covariance. Effect of group (HC, PD) and covariates (age, sex at birth and education) on EAR performance (ERI, EAI).

	F	df	*p*
Bivariate tests			
Group	3.400	2, 34	.045*
Age	15.66	2, 34	<.001*
Sex at birth	4.740	2, 34	.015*
Education	1.030	2, 34	.367
Univariate tests			
ERI			
Group	3.982	1	.054
Age	28.168	1	<.001*
Sex at birth	64.87	1	.121
Education	50.21	1	.171
EAI			
Group	6.320	1	.017*
Age	19.867	1	<.001*
Sex at birth	211.43	1	.004*
Education	2.82	1	.720

Abbreviations: EAI, emotion authenticity index; EAR, emotion recognition test; ERI, emotion recognition index; HC, healthy control subjects; PD, individuals with Parkinson's disease.

* *p* values <.05.

When having to recognise the genuineness of emotions, PD patients are more likely to consider authentic expressions actually simulated than vice versa. In the authenticity recognition, PD patients got 27.42% of the answers wrong; of the errors, 56.23% were failure to recognise simulation, 43.77% were failure to recognise genuineness. Similar behaviour was shown by HC, who got 17% of the answers wrong; of the errors, 54.41% were failure to recognise the simulation, 45.59% were failure to recognise genuineness.

The mean number of correct answers given by PD patients and HC for each type of emotion, when asked to identify it, is shown in Table 5. In PD patients, the emotion of fear is the least recognised (4.6 ± 1.93 of 10), followed by anger (6.05 ± 1.54 of 10). This is also observed in HC (fear: 5.43 ± 1.45 of 10, anger: 6.57 ± 2.65 of 10).

**TABLE 5:** Mean values ± standard deviation of individual emotion recognition index for each emotion achieved by individuals with Parkinson's disease and healthy control subjects.

	HC	PD
Surprise	9.64 ± .63	9.25 ± 1.29
Happiness	8.36 ± 1.28	8.75 ± 1.25
Sadness	8.14 ± 1.83	7.85 ± 2.34
Disgust	8.36 ± 1.08	7.65 ± 1.95
Anger	6.57 ± 2.65	6.05 ± 1.54
Fear	5.43 ± 1.45	4.60 ± 1.93

Abbreviations: HC, healthy control subjects; PD, individuals with Parkinson's disease.

The mean number of correct answers given by PD patients and HC for each type of emotion, when asked to identify the authenticity of emotion, is shown in Table 6. In this case, performance appears to be consistent within each group.

**TABLE 6:** Mean values ± standard deviation of individual authenticity recognition index for each emotion achieved by individuals with Parkinson's disease and healthy control subjects.

	HC	PD
Surprise	7.87	6.15
Happiness	8.20	8.00
Sadness	8.47	7.55
Disgust	7.87	7.40
Anger	7.73	7.40
Fear	7.13	6.80

Abbreviations: HC, healthy control subjects; PD, individuals with Parkinson's disease.

The effects of clinical and cognitive variables on emotion recognition are shown in Table 7. For both variables expressing emotion recognition (ERI, EAI), there were no significant main effects of disease duration (ERI: F = .412, *p* = .53; EAI: F = 3.808, *p* = .071), LEDD (ERI: F = .557, *p* = .468; EAI: F = .962, *p* = .343) and MoCA (ERI: F = .147, *p* = .708; EAI: F = 4.074, *p* = .063). ERI is significantly affected by BFRT (F = 8.287, *p* = .01) and UPDRS‐III (F = 6.079, *p* = .03), while for EAI the effect of BFRT (F = 4.074, *p* = .06) and UPDRS‐III (F = 6.69, *p* = .43) does not reach significance.

**TABLE 7:** Effects of clinical and cognitive variables on emotion and authenticity recognition.

	ERI (correct score)		EAI (correct score)	
Disease duration (years)	F = .412	*p* = .53	F = 3.808	*p* = .07
UPDRS‐III	F = 6.079	*p* = .03*	F = .669	*p* = .43
LEDD (mg)	F = .557	*p* = .47	F = .962	*p* = .34
MoCA (correct score)	F = .147	*p* = .71	F = .300	*p* = .59
BFRT (correct score)	F = 8.287	*p* = .01*	F = 4.074	*p* = .06

Abbreviations: BFRT, Benton Facial Recognition Test; EAI, emotion authenticity recognition; ERI, emotion recognition index; LEDD, Levodopa Equivalent Daily Dose; MoCA, Montreal Cognitive Assessment; UPDRS‐III, Unified Parkinson's Disease Rating‐Scale‐third section.

* *p* values ≤.05.

## DISCUSSION

In the facial emotion recognition process, the predominant defect of PD patients concerns discrimination of the genuineness of the expression. The ability to typify emotions is not impaired, although most of the studies indicate a PD impairment. (Argaud et al., 2018; Coundouris et al., 2020; Gray & Tickle‐Degnen, 2010; Péron et al., 2012). The clinical and cognitive characteristics of our sample and the specific features of the EAR test may explain this inconsistency in the results. Patients with severe PD, cognitive impairment or visual‐perceptual difficulties were excluded from our study; this reduces the possibility of a drop in performance compared to controls. Furthermore, the stimuli of the EAR test consist of intensely and dynamically expressed emotions, which are more corresponding to real life than those of classical tests; this makes our test easier, and, above all, facilitates the use of the perceptual‐semantic strategy, which is unlikely to be compromised in the PD patients we selected.

However, a dissociation emerged between the recognition of emotions and their authenticity that needs to be confirmed by subsequent studies on the question. In fact, although one of the most important aspects of communication and social interaction lies in the perception of the authenticity of the emotion expressed, this topic has remained largely unexplored, and it is not known whether this capacity is impaired in neurological and psychiatric illnesses.

Classification of emotional expressions into basic categories is usually achieved through perceptual analysis of observed faces (Niedenthal et al., 2010), which allows the identification of distinctive salient features (Smith et al., 2005). At the neural level, this process appears to be supported by the occipitotemporal cortices (Adolphs, 2002). In line with other studies (Calvo & Lundqvist, 2008; Czernecki et al., 2021; Dodich et al., 2022; Mattavelli et al., 2021; Scarpazza et al., 2024; Tracy & Robins, 2008), we found that in both HC and PD patients the greatest difficulty concerns fear. It is believed (Roy‐Charland et al., 2014) that the expression of fear is difficult to discriminate perceptually because it is realised by muscle configurations similar to those of other emotions, such as surprise (Gagnon et al., 2010), towards which observers direct their responses because they are less socially threatening emotions (Jack et al., 2009; Scarpazza et al., 2024).

While the analysis of visual facial features may be sufficient to classify prototypical expressions, this process is unlikely to be sufficient to recognise less prototypical emotional expressions or to represent their subtle meanings (Niedenthal et al., 2010), or even to distinguish their authenticity (Scarpazza et al., 2024). In these cases, non‐visual conceptual information about the emotion (i.e., knowledge about the expresser's emotional reactions and the social situation he or she is experiencing) is hypothesised to be supportive (Halberstadt et al., 2009; Halberstadt & Niedenthal, 2001; Hess et al., 2009), but embodied simulation could play an important role (Decety & Chaminade, 2003; Gallese, 2003; Goldman & Sripada, 2005; Niedenthal et al., 2005, 2007; Winkielman et al., 2009).

We hypothesise that, if the observed facial expression is clear (i.e., sufficiently intense and not confused), the identification of emotion occurs mainly through the analysis of the morphological and geometrical properties of the visual stimulus, or through the recollection of situations with which the facial expression has been associated in the past, independent of emotional reactivation. These processes occur through extra‐limbic circuits and are used by the PD patients to compensate for the dysfunction of the limbic orbito‐mesial extrapyramidal circuit, which may precede dorsolateral cognitive circuit involvement in disease progression. As limbic dysfunction is compensated by cognitive processes, it does not become evident until after the onset of cognitive impairment, although it may precede it (Herrera et al., 2011).

However, when it is not a question of identifying the type of emotion, but its authenticity, one would mainly rely on embodied cognition, that is, on the activation with a mirror mechanism of the same motor reaction presented by the model. If so, the dysfunction of the limbic orbito‐mesial extrapyramidal in PD would compromise the mirror mechanism and the ability to distinguish the authenticity of emotions.

Our results provide support for the hypothesis that facial emotion recognition is not mainly based on embodied cognition. Internal simulation mechanisms and the role of the mirror neuron system are best expressed by the ability to recognise the genuineness of emotional expressions, the latter being more impaired in PD patients.

As for the effects of clinical variables on EAR performance, the absence of significance could be due to the low power of the analysis, linked to the small sample size. Furthermore, it must be considered that only patients with mild or moderate motor symptoms, often in the early stages of the disease, were selected; this in every case limits the generalisation of the result.

Similarly, global cognitive efficiency assessed with MoCA also does not seem to influence performance at the EAR. Again, the risk of type II error is high and only patients with normal MoCA scores were enrolled. Interestingly, patients' visual‐perceptual abilities influence emotion recognition, a fact already known in the literature (Alonso‐Recio et al., 2014; Dujardin et al., 2013; Marneweck et al., 2014; Narme et al., 2011), but not the authenticity recognition.

## CONCLUSION

In conclusion, our results support the hypothesis that facial emotion recognition occurs simultaneously through two mechanisms, embodied simulation and visual‐perceptual processing, and that under pathological conditions one can supplant the other either more or less efficiently. Instead, embodied simulation would play a primary role in recognising the genuineness of emotional expressions. As a matter of fact, the impairment of PD patients mainly concerns the ability to distinguish the emotion authenticity, which would almost entirely require embodied simulation and mirror system activation (Niedenthal et al., 2010). On the other hand, the ability to identify the meaning of emotional facial expressions seems to be maintained, as PD patients would rely on voluntary recognition mechanisms instead of the embodied cognition (Folstein et al., 2013). The disturbance of authenticity recognition in PD patients can be present even in the absence of cognitive impairment and depends on a malfunction of the extrapyramidal limbic circuit.

We consider our study to be innovative for its subject matter. Alterations in social behaviour are often the direct consequence of misinterpretation of emotions. Indeed, emotional expressions do not always reflect true feelings (Durán & Fernández‐Dols, 2021); individuals may deliberately simulate emotions without a corresponding authentic context in order to gain strategic advantages (Monaro et al., 2022). Trading false emotions for authentic ones can have negative outcomes for the perceiver and interfere with social relationships. Furthermore, a possible inability to recognise the authenticity of emotions could explain why individuals with psychiatric and neurological disorders often have difficulties in social engagement (Henry et al., 2016; Meyer‐Lindenberg & Tost, 2012). In PD, this impairment could reveal the dysfunction of the extrapyramidal orbito‐mesial limbic circuit, which could precede the involvement of the dorsolateral cognitive circuit in disease progression.

Our study should be considered a pilot study. Despite the limitations resulting from the small sample size, the lax patient selection criteria (no cognitive tests or additional psychological questionnaires) and the lack of individual performance analysis for individual emotions, the results are consistent with the hypothesis and encourage further validation studies.

## AUTHOR CONTRIBUTIONS

**Agnese Anzani:** Conceptualization; data curation; investigation; writing – original draft; writing – review and editing; visualization. **Stefano Zago:** Conceptualization; investigation. Teresa Difonzo: Conceptualization, investigation. **Cristina Scarpazza:** Conceptualization, investigation, writing – original draft; resources. **Nadia Bolognini:** Conceptualization, investigation, writing – original draft. **Giulia Franco:** Conceptualization, investigation. **Alessio Di Fonzo:** Conceptualization, investigation. **Maria Cristina Saetti:** Conceptualization, methodology, supervision, formal analysis, writing – original draft, writing – review and editing, visualization, project administration, software.

## CONFLICT OF INTEREST STATEMENT

The authors declare no conflicts of interest.

## Data Availability

Datasets cannot be made publicly available on both ethical and legal grounds but may be made available upon reasonable request of interested researchers to the Corresponding Author, who will in turn forward a request for a data transfer agreement to the relevant Ethical Committee.
